# Quality of mental health care for forcibly displaced children and adolescents in the WHO European region: A scoping review of barriers and facilitators

**DOI:** 10.1007/s00787-025-02833-3

**Published:** 2025-08-27

**Authors:** Lars Dumke, Shobhana Nagraj, Salma Yusuf, Hanan Abukmail, Erva Nur Cinar, Mohammad S. Razai, Gemma Whyatt, Jennifer Hall, Joao Breda, Anastasia Giannaki, Florian Scharpf, Malte Behrendt, Ingo Schäfer, Eleanor Chatburn

**Affiliations:** 1 Department of Psychiatry and Psychotherapy, University Medical Center Hamburg-Eppendorf, Hamburg, Germany; 2 Department of Public Health and Primary Care, University of Cambridge, Cambridge, UK; 3 East London NHS Foundation Trust, London, UK; 4 International Health System Research group, Department of Engineering, University of Cambridge, Cambridge, UK; 5 Faculty of Epidemiology and Population Health, London School of Hygiene and Tropical Medicine, London, WC1E 7HT UK; 6 Guy’s and St Thomas’ NHS Foundation Trust, London, UK; 7 WHO Athens Office on Quality of Care and Patient Safety, WHO Regional Office for Europe, Athens, Greece; 8 Department of Psychology, Bielefeld University, Bielefeld, Germany; 9 Department of Clinical Psychology and Psychological Therapies, Norwich Medical School, University of East Anglia, Norwich, UK

**Keywords:** Barriers, Access, Mental health, Children, Youth, Refugees, Migrants, Quality

## Abstract

Forcibly displaced children and adolescents in the World Health Organization (WHO) European Region have high mental health needs, yet few manage to access mental health services, and those who do may encounter inadequate care. This scoping review aimed to identify and synthesize the available evidence on barriers and facilitators to quality mental health care for forcibly displaced children and adolescents in the WHO European Region. We applied the PRISMA guideline extension for scoping reviews, searching five scholarly databases and grey literature published between 2004 and 2025. A total of 7,327 records were screened, with 18 articles included. We used the WHO Quality Standards for Child and Youth Mental Health Services as an analytical framework to map the evidence. The identified studies represented only 7 out of 53 countries in the WHO European Region. Most studies employed qualitative research designs, with a lack of quantitative evidence using quality indicators. Available evidence revealed multiple, intersecting barriers to quality mental health care, including restrictive health care policies, service constraints and a lack of provider competence. Facilitators included the presence of community-based support, networks of interconnected services, task shifting and evidence-based scalable interventions. Major gaps remain in both the data and in the methods used to generate evidence for improving quality of mental health care for forcibly displaced children and adolescents in the WHO European Region. Our review highlighted the discrepancy between the care provided and the recommended WHO Quality Standards for Child and Youth Mental Health Services. There is a need for more targeted efforts to assess and improve the quality of mental health care for forcibly displaced children and adolescents.

**Supplementary Information:**

The online version contains supplementary material available at 10.1007/s00787-025-02833-3.

Children and adolescents face a high burden of mental ill health [1]. In the World Health Organization (WHO) European Region, youth mental health has deteriorated over the past two decades, posing a major threat to well-being and psychosocial functioning [1, 2]. It is estimated that one in five young people live with a mental health condition [3]. The risk of developing mental health problems is particularly high among marginalized populations affected by multiple social and structural determinants of health, such as children and adolescents forcibly displaced due to persecution, conflict, violence and human rights violations^1^ [4]. With more than 9 million forcibly displaced children and adolescents (FDCA) in the 53 countries of the WHO European Region, including major host countries such as Turkey and Germany, strengthening mental health services to adequately meet their needs has become an urgent public health priority [4].

FDCA face accumulating risk factors before, during and after their migration. These include individual, family, community and sociocultural level stressors such as war-related trauma, perilous migration journeys, separation from immediate family, uncertain asylum procedures, and discrimination [5]. In Europe, an estimated one in three FDCA has a mental health condition requiring treatment [6], with prevalence rates of post-traumatic stress disorder (PTSD), depression, anxiety disorders, and attention-deficit/hyperactivity disorder (ADHD) exceeding those among non-displaced peers in host countries [7]. Without adequate treatment, mental health problems that occur in childhood and adolescence often persist for years, with serious consequences throughout the life course [8]. These may include educational disadvantages, difficulties integrating into a new environment, and lasting susceptibility to poor mental and physical health [5, 8]. The increased rates of mental health problems and their long-term consequences underline the need for accessible and quality mental health treatment for FDCA.

However, there are serious deficiencies in mental health care for children and adolescents in the WHO European Region. Among the general population of young people in Europe, one in two report unmet mental health needs [9]. This treatment gap is even more acute for FDCA, who have disproportionately lower treatment rates compared to their non-displaced peers [10]. Broader shortcomings in the mental health system - such as limited availability of services and long waiting times - affect all children and adolescents in Europe. However, the markedly lower treatment rates for FDCA point to systemic and structural barriers hindering their access to care. Moreover, forcibly displaced populations are at risk of receiving inequitable and potentially lower quality care [10, 11]. For example, they may receive different treatment recommendations and fewer sessions in outpatient care than the host population [11–13]. Despite these critical issues, comprehensive and current data on the barriers and facilitators to quality mental health services for FDCA in the WHO European Region remains limited.

To our knowledge, only one review has comprehensively analysed mental health service use by children and young people with a refugee background [14]. There is a need for a detailed updated review because of the three-fold increase in forcibly displaced people in Europe in the past 10 years [15]. Recent reviews have either focused on broader migrant youth populations or the perceptions of specific subgroups (e.g., unaccompanied refugee minors) [16, 17]. No review has brought together the perspectives of children and adolescents, service providers, and service coordinators and managers. Integrating all stakeholder perspectives can provide a more holistic perspective considering both demand- and supply-side factors [18].

In addition to measuring access to care, it is essential to also map the quality of mental health care for FDCA to inform policy and practice. According to the WHO, quality of mental health care refers to the extent to which services produce the desired mental health outcomes and adhere to evidence-based practice [19]. The WHO recently introduced a comprehensive framework outlining quality standards for child and adolescent mental health services in the European Region [19]. This framework provides a timely basis for examining the barriers and facilitators to quality mental health care for FDCA. Recent reviews have shown that displaced populations face a wide range of barriers to receiving adequate health care, including general health system barriers as well as distinct barriers closely linked to legal, structural, and sociocultural aspects of displacement [18, 20]. However, no review to date has synthesized the evidence on quality of mental health care specifically for FDCA, and the scope of the literature and existing knowledge gaps remain unclear. Addressing this gap by systematically identifying and mapping the available evidence could help inform the design and evaluation of strategies to improve the quality of mental health care for this population.

The aim of this scoping review is to synthesize the available evidence on the barriers and facilitators to quality mental health care for FDCA in the WHO European Region. Guided by the WHO Quality Standards for Child and Youth Mental Health Services, we will systematically identify research gaps and provide evidence-informed recommendations for improving the quality of mental health care for FDCA in Europe.

## Methods

This scoping review followed the PRISMA Extension for Scoping Reviews (PRISMA-ScR) [21]. Given the lack of prior comprehensive reviews on this topic and the anticipated heterogeneity, a scoping review methodology was deemed appropriate. A pre-registered protocol (10.17605/OSF.IO/AK74F) is available. No changes were made to the protocol.

Our research question was “*What are the relevant barriers and facilitators to quality mental health care for forcibly displaced children and adolescents in the WHO European Region*?”.

### Eligibility criteria

Inclusion criteria were: (1) quantitative, qualitative, and mixed-method research exploring barriers and facilitators to quality mental health care as defined by the WHO; (2) studies that involved FDCA (0–19 years old as defined by the WHO), their families, caregivers, mental health care providers, mental health service managers, commissioners, policymakers, humanitarian actors as participants; (3) research conducted within the WHO European Region.

Studies were excluded if they: (1) did not specifically focus on mental health care; (2) did not explicitly examine barriers and facilitators to quality mental health care; (3) focused on forcibly displaced adults or on the general migrant population without clearly specifying findings for FDCA; (4) were of a publication type that did not present original empirical data (e.g., editorials, commentaries, conference abstracts, reviews, and book chapters).

### Search strategy

Details of the search strategy are outlined in the protocol. The following five databases were searched to identify relevant articles: Embase (via Elsevier), Medline (via PubMed), PsycINFO (via EBSCOhost), Scopus (via Elsevier), and Web of Science. A tailored search strategy using the population, concept, context framework was employed and adapted for each database. Additional sources were searched through citation-chaining and grey literature searches including documents from libraries, repositories, and websites of national and international NGOs and academic institutions. Literature published in English or German in the last 20 years (since 2004) was considered for inclusion.

### Study selection

**Fig. 1:**
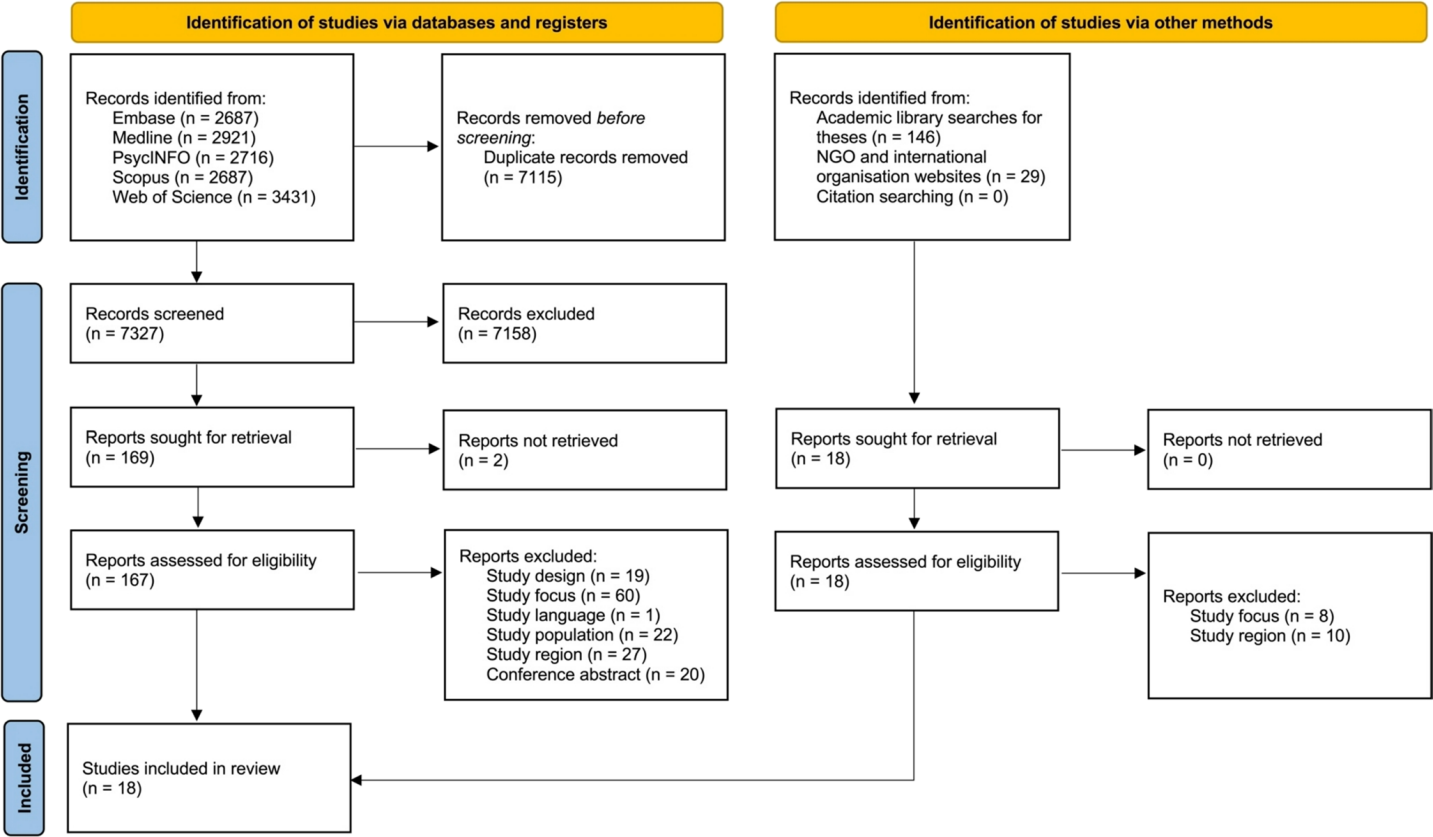
PRISMA Flowchart

Figure 1 shows the PRISMA flow diagram. During the screening and full-text assessment phase, each title was assessed independently by two reviewers. A third reviewer resolved any disagreements between the reviewers.

### Data extraction

Data were extracted with a piloted data extraction form. The following information was extracted from included studies: study name, year of publication, study aims and methods, participants, context, and key findings on barriers and facilitators to quality mental health care. There were no ad-hoc modifications to the data extraction form described in the protocol.

### Data analysis and presentation

A narrative synthesis was conducted to summarize findings on barriers and facilitators to quality mental health care. This was guided by the WHO Quality Standards for Child and Youth Mental Health Services [19], taking into account the different domains of quality (i.e., participation and empowerment, rights and safety, family and community engagement, smooth transitions, timely support, developmentally and evidence-based, competent and appropriate workforce, data collection and quality improvement) where barriers and facilitators can occur. Definitions of the WHO quality domains are provided in Online Resource 1.

## Results

### Characteristics of included studies

A total of 18 peer-reviewed studies were included. Table 1 provides a detailed overview of these studies. The studies were conducted in seven countries in the WHO European Region, with 15 studies in Western European countries (eight from the UK) and four in Turkey.

**Table 1:** Summary of studies included in the scoping review

Author, year	Country	Aim of the study	Study design (and methods)	Sample characteristics		Setting
				Forcibly displaced children and youth	Other participant groups	
Bleile et al. [22]	Netherlands	To evaluate the implementation of the TeamUp intervention for refugee children, in order to inform future development, evaluation and scaling.	Mixed methods (Cross-sectional surveys, observation checklists, focus group discussions, and key informant interviews)	Focus group discussion: *N* = 79 children 5–17 years 29 female, 50 male Key informant interviews: *N* = 15 7–15 years, 3 female, 12 male	Focus group discussion: *N* = 24 service providers of the intervention Key informant interviews: *N* = 10 staff members of the asylum seekers centers Cross-sectional survey: *N* = 99 service providers of the intervention Observation checklist: *N* = 81 service providers of the intervention	Refugee reception centers implementing a psychosocial intervention (‘TeamUp’) for refugee children
Eruyar et al. [23]	Turkey, UK	To understand the responsiveness of support systems for refugee young people with mental health needs across two countries, Turkey and the UK, from the perspectives of young refugees, parents, and professionals.	Qualitative (Focus group discussions)	*N* = 19 In Turkey: 10 children 10–17 years 2 female, 8 male from Syria living with parents In UK: 9 children 16–18 years 1 female, 8 male from Syria unaccompanied minors	*N* = 28 In Turkey: 10 carers (3 mothers, 7 fathers) 7 service providers (2 teachers, 3 clinical psychologist, 2 social workers) In UK: 7 carers (7 mothers) 4 service providers (1 general health practitioners, 1 mental health practitioner, 1 educational psychologist, 1 NGO worker)	Mental health services in the general health system
Fazel [24]	UK	To describe the role of schools in supporting the overall development of refugee children and the importance of peer interactions.	Qualitative (Semi-structured interviews)	*N* = 40 15–24 years, Median: 17 years 11 female, 29 male from 20 different countries 13 unaccompanied 6 months-7 years in UK	N/A	School-based mental health services
Fazel et al. [25]	UK	To address the absence of data on the actual experience of adolescents directly seen by services by asking those seen by school-based mental health services about their experience of being seen within the school location, how they perceived the therapy, whether or not it helped them and finally about any worries and preoccupations that might be impacting their time at school.	Qualitative (Semi-structured interviews)	*N* = 40 15–24 years, Median: 17 years 11 female, 29 male from 20 different countries 13 unaccompanied 6 months-7 years in UK	N/A	School-based mental health services
Jarlby et al. [26]	Denmark	To explore how unaccompanied refugee adolescents perceive mental health and the mental healthcare they are provided with, and which services they believe would benefit them.	Qualitative (Semi-structured individual interviews, focus group interview)	*N* = 6 17–18 years 0 female, 6 male 3 from the Middle East, 3 from Southeast Asia 2–3 years in Denmark	*N* = 6 6 service providers (4 social workers, 1 caseworker, 1 paediatrician)	Mental health services in the general health system
Lampa et al. [27]	Sweden	To evaluate the factors and agents that have facilitated the implementation and maintenance of ‘Teaching Recovery Techniques’, a community-based intervention for refugee youth reporting symptoms of post-traumatic stress.	Qualitative (Semi-structured interviews)	N/A	*N* = 7 6 service providers (4 social workers, 1 manager	Health care services implementing a community-based intervention (‘Teaching Recovery Techniques’) for refugee youth
Majumder et al. [28]	UK	To appreciate the views and perceptions that unaccompanied minors hold about mental health and services.	Qualitative (Semi-structured interviews)	*N* = 15 15–18 years 1 female, 14 male 11 from Afghanistan, 2 Iran, 1 Eritrea, 1 Somalia	N/A	Mental health services in the general health system
Majumder et al. [29]	UK	To examine mental health services from the perspective of unaccompanied refugee minors and their carers.	Qualitative (Semi-structured interviews)	*N* = 15 15–18 years 1 female, 14 male 11 from Afghanistan, 2 Iran, 1 Eritrea, 1 Somalia	*N* = 17 17 carers (12 foster carers, 4 residential carers, 1 social worker)	Mental health services in the general health system
Majumder [30]	UK	To explore the beliefs and perceptions of unaccompanied refugee children in the context of mental illness, its treatment, service engagement and the stigma attached to it.	Qualitative (Semi-structured interviews)	*N* = 15 15–18 years 1 female, 14 male 11 from Afghanistan, 2 Iran, 1 Eritrea, 1 Somalia	*N* = 17 17 carers (12 foster carers, 4 residential carers, 1 social worker)	Mental health services in the general health system
Michelson and Sclare [31]	UK	To compare patterns of service utilization between UAMs and accompanied children at a youth refugee mental health service and to compare the clinical strategies undertaken by team members in addressing the respective psychological needs.	Quantitative (Cross sectional design)	*N* = 78 49 unaccompanied minors Mean age: 16.29 years 28 female, 21 male from 15 different countries, most participants from African countries 29 accompanied minors Mean age: 10.97 years 16 female, 13 male from 25 different countries, most participants from African countries	N/A	Specialist mental health service for young refugees
Namer et al. [32]	Germany	To describe asylum seeking and refugee adolescents’ mental health needs and psychotherapy utilization (1); to identify the predictors of their mental health-related help-seeking patterns (2); and to contextualize mental healthcare provision from mental health professionals’ perspectives (3).	Mixed methods (Cross-sectional survey, semi-structured interviews)	*N* = 216 11–18 years, mean age: 14.66 years 98 female, 111 male 101 from Syria, 54 from Iraq, 25 from Afghanistan, 36 from other countries	*N* = 9 9 service providers (4 child psychotherapists, 1 social worker, 4 school psychologists)	Mental health services in the general health system
Poyraz Fındıket al. [33]	Turkey	To examine psychiatric service utilization patterns and clinical profiles of refugee children and compare them to native children within the same age group who applied to the same child and adolescent psychiatry service.	Quantitative (Comparative research design)	*N* = 91 mean age: 9.02 years 29 female, 62 male from Syria 59 children resettled for more than 36 months (+ 82 non-refugee children)	N/A	Child and adolescent psychiatry service in the regular mental health system
Seyda et al. [28]	Turkey	To understand the process of designing service plans to improve mental health provision for refugee children and young people in Turkey, according to a trauma-­informed collaborative framework	Qualitative (Participatory focus groups)	N/A	*N =* 14 12 service providers (6 psychologists, 3 social workers, 3 physiotherapists) 2 service managers/coordinators	Refugee mental health services offered by NGOs
Sualp et al. [34]	Turkey	What are the needs of refugees and MHPs to conduct more effective group work interventions with refugee children?	Qualitative (Semi-structured interviews)	N/A	*N* = 10 10 service providers (4 social workers, 5 psychologists, 1 psychiatrist)	Refugee mental health services offering group interventions for refugee children
Van Es et al. [35]	Netherlands	To explore the main request for help among URMs in the Netherlands referred to a multimodal trauma-focused treatment approach, the treatment integrity and feasibility, and the course of symptoms of PTSD and/or depression.	Mixed methods (Cross-sectional survey including open-ended questions)	*N* = 41 12–19 years, mean age: 16.5 years 14 female, 27 male 31 from Eritrea, 9 from Syria, 1 from Afghanistan	*N* = 17 17 service providers (9 therapists, 8 intercultural mediators)	Multimodal trauma-focused treatment approach performed by therapists working at various mental health care institutions
Van Es et al. [36]	Netherlands	To provide an initial indication of the effectiveness of this multimodal trauma-focused approach for traumatized URMs (1); to provide a qualitative evaluation assessing treatment satisfaction of the participating URMs (2).	Mixed methods (longitudinal survey, qualitative interviews questions)	*N* = 10 15–18 years, mean age: 16.5 2 female, 8 male 9 from Eritrea, 1 from Syria	N/A	Multimodal trauma-focused treatment approach performed by therapists working at various mental health care institutions
Versteele et al. [37]	Belgium	How does a collaborative approach to promoting access to mental health care for URMs allow for working with the interplay between cultural and structural determinants of mental health and traumatic suffering?	Qualitative (Qualitative interviews, case documents)	*N* = 1 16 years male from Afghanistan unaccompanied minor (+ case review of 10 cases: 15–17 years all male, unaccompanied from Afghanistan)	*N* = 5 4 service providers (2 clinical psychologists, 1 cultural mediator, 1 child psychiatrist) 1 carer (1 legal guardian)	Collaborative mental health care approach in a psychiatric day clinic
Yim et al. [38]	UK	To review existing recommendations on psychosocial interventions to address UASC mental health needs (1); to examine the current mental health provision within health and social care in England from service providers’ perspectives (2); to synthesise recommendations and shortcomings in the current approaches to reduce health inequity in statutory settings (3).	Qualitative (Qualitative interviews, literature and guidelines review)	N/A	*N* = 15 12 service providers (10 clinical psychologists or psychotherapists, 2 social workers) 3 service managers/coordinators (1 service manager, 2 health and social care commissioners)	Statutory mental health services

*Notes. ASR* asylum seekers and refugees, *MHP* Mental Health Professionals, *UAM* Unaccompanied Minors, *UASC* Unaccompanied Asylum Seeking Children, *URM* Unaccompanied Refugee Minors, *PTSD* Post Traumatic Stress Disorder

Of the 18 included studies, 12 employed qualitative methods including semi-structured interviews (10 studies), focus group discussions (3 studies), and/or case documents and clinic guidelines analysis (2 studies). Two studies used quantitative methods to compare service utilisation patterns, and four applied mixed-methods design, combining cross-sectional surveys with qualitative interviews.

Nine studies focused on regular mental health care services, two studies explored school-based mental health services, three examined the implementation of specific psychosocial interventions, and another four studies described the implementation of specific care models.

Regarding participant groups, eight studies included both children and adolescents and at least one other participant group. Six studies included only FDCA and four studies included only other participant groups. Among the 14 studies that included FDCA, seven studies focused on unaccompanied minors. The sample size of FDCA ranged from 1 to 216. Ages ranged from 5 to 24 years, with participants mainly from Syria, Afghanistan, Iraq, Somalia, Eritrea, and Iran. Length of stay varied from six months to seven years. Gender distribution was predominantly male, with only one study reporting a female majority.

Among the 12 studies that included other participant groups, five studies included carers (parents, foster carers, residential care home staff, legal guardian), ten studies included mental health service providers (including clinical psychologists, psychotherapists, child psychiatrists, social workers, school psychologists), and three studies included service coordinators, managers or commissioners.

### Quality status of mental health care for forcibly displaced children and adolescents

Table 2 provides an overview of the quality domains covered by each study. The most frequently discussed quality domains were participation and empowerment, rights and safety, and family and community engagement. Few studies discussed data collection, quality improvement, and developmentally appropriate and evidence-based care. Table 3 outlines the main barriers and facilitators for each domain, with the narrative synthesis presented below.

**Table 2:** Quality domains from the WHO quality standards for child and youth mental health services discussed by the included studies

Study author, year	Participation & Empowerment	Rights & Safety	Family and community engagement	Smooth Transitions	Timely Support	Developmentally Appropriate and Evidence-based	Competent and Appropriate Workforce	Quality improvement and data collection
Bleile et al. [22]	X	X				X	X	
Eruyar et al. [23]		X	X	X	X			
Fazel [24]		X	X					
Fazel et al. [25]	X		X					
Jarlby et al. [26]	X	X				X	X	
Lampa et al. [27]		X	X	X	X			
Majumder et al. [28]	X	X						
Majumder et al. [29]	X	X					X	
Majumder [30]	X	X						
Michelson and Sclare [31]				X	X	X		
Namer et al. [32]		X	X	X	X		X	
Poyraz Fındık [33]				X				
Seyda et al. [28]	X		X	X	X			X
Sualp et al. [34]		X	X	X	X		X	
Van Es et al. [35]	X	X	X					X
Van Es et al. [36]	X	X	X			X		
Versteele et al. [37]	X	X	X	X			X	
Yim et al. [29]			X	X	X	X	X	X

**Table 3:** Summary of identified barriers and facilitators to quality mental health care mapped on the quality domains outlined in the WHO European region quality standards for child and youth mental health services

Quality domain	Barriers	Facilitators
Participation & empowerment	● Lack of individualized and co-developed care ● Disregard for children’s and adolescents’ perspectives and (cultural) understandings in treatment ● Rigid and inflexible therapeutic approaches	● Establishing shared therapeutic goals ● Using flexible, needs-based treatment approaches ● Implementing community awareness campaigns ● Making use of peer support approaches
Rights & safety	● Legal restrictions ● Inadequate informed consent ● Medicalized concepts of mental health services ● Language and interpretation challenges ● Discrimination	● Minding mental health stigma ● Using culturally sensitive informed consent ● Taking adequate time for psychoeducation ● Providing interpreter training and supervision
Family and community engagement	● Lack of mental health awareness ● Mental health stigma ● Lack of coordination between services	● Strengthening school involvement ● Organising regular network meetings ● Implementing peer support, community-based, and mobile services
Smooth transitions	● Fragmented services ● Uncoordinated referral pathways ● Service provider’s limited awareness of available referral services ● High eligibility thresholds for referrals ● Administrative barriers (e.g., cost coverage of treatment) ● Asylum uncertainties	● Establishing coordinating agencies ● Networking to build inter-agency collaborations (e.g., disseminating information, setting up network meetings) ● Providing training for referral institutions (e.g., about trauma-informed care) ● Providing clear and accessible referral information ● Setting up structured follow-up strategies
Timely support	● Legal restrictions ● Asylum uncertainties ● Service constraints in regular and specialised mental health care ● Inadequate resources and funding for child and adolescent refugee mental health services	● Implementing scalable psychosocial interventions ● Task-shifting approaches
Developmentally and evidence-based	● Service provider’s discomfort with trauma-focused treatment ● Lack of support system in accommodations ● Different age groups and needs	● Integrating social, sports, and arts activities into mental health care
Competent and appropriate workforce	● Staff shortages ● High staff turnover ● Lack of supervision ● Lack of cultural competence ● Lack of diversity in workforce	● Training and ongoing supervision for service providers on mental health problems in refugee populations ● Training and ongoing supervision for service providers on cultural competency and anti-discriminatory practice ● Training for service providers on self-care strategies ● More diverse workforce
Quality improvement and data collection	● Time constraints in services ● Service providers’ concerns about data collection ● Lack of clearly defined indicators ● Gaps in healthcare databases	● Defining and standardizing measurable indicators for service planning

#### Participation and empowerment

Studies reported a lack of individualised and co-developed care that takes into account children and adolescents’ treatment preferences and goals [25, 26, 29]. Children and adolescents described feeling patronised, infantilised, and talked down to during treatment [26, 29]. Treatment was seen as disconnected from their lived experiences, focusing on secondary issues that were not considered a priority, instead of immediate concerns and difficulties in their lives, such as asylum uncertainty, difficulties at school, or social exclusion [25, 26, 28, 29].

Jarlby et al. [26] found that mental health services often neglected youth’s understandings of mental health, as well as the political and structural aspects of their current life situations. The lack of engagement with their perspectives and treatment wishes led to reluctance to attend treatment, passive compliance and disengagement from the therapeutic process [28, 29]. In addition, some children and young people raised concerns about rigid and repetitive questioning in therapy, which they perceived as unhelpful and uncomfortable [25, 26, 28]. Although in some cases the therapeutic approach exacerbated rather than alleviated distress, young people’s feedback about the unpleasantness of the therapeutic process was dismissed as a cultural difference in understanding mental health care [26, 28, 29].

Several facilitators were identified that fostered the participation and empowerment in mental health services. This included establishing shared therapeutic goals, facilitated by adequate time for building trust, psychoeducation and explanation of interventions [35, 37]. A flexible approach to treatment planning was recommended to respond to current and practical needs as they arise [33, 35–37]. Furthermore, actively involving children and caregivers in awareness campaigns targeted at various stakeholders (e.g., community, teachers, professionals, and policy makers) was considered important to improve patient engagement and inclusion in the health care system [33]. In addition, the use of peers was suggested by service providers as useful in raising awareness and encouraging others to engage with mental health services [22].

#### Rights and safety

Informed consent procedures in services were reported to be inadequate. Young people did not always understand the risks and benefits of treatment and alternative care options, as evidenced in descriptions of youths’ past experiences in trauma-focused therapy in two studies [26, 29]. In addition, informed consent around the use of medication was found to be insufficient, with children and adolescents not being fully informed about the reasons for medication and its appropriate use [28].

Another barrier was the stigma around mental health care, particularly where services represented a medicalised concept of mental health [30, 33]. Majumder [30] describes how some youth denied having mental health problems, despite having accessed a mental health service. The absence of a shared language around mental health, combined with young people’s reluctance to discuss or acknowledge symptoms were seen as barriers to quality care by service providers [30].

Language challenges also raised issues of rights and safety. Service providers described a lack of established policies and procedures, particularly in relation to cost coverage, to ensure professional language interpretation [23, 32]. Due to a lack of trained interpreters and financial resources for formal interpreting services, many mental health services have to rely on community or family members, leading to serious concerns about confidentiality and fears of stigmatising consequences of seeking help [22, 23, 32, 35]. While service providers described the use of interpreting services and intercultural mediators as beneficial for understanding culture and building strong therapeutic relationships, problems were described in cases when interpreters joined online or over the phone [35].

Several studies reported that young people are often not treated with dignity and respect in mental health services. Refugee youth described being treated as outsiders and experiencing discrimination in mental health care services [26, 28, 29]. The lack of diverse representation within the mental health workforce further exacerbated feelings of alienation and discomfort in mental health services (Majumder, 2018).

Uncertainties about statutory child protection measures and asylum procedures, as well as legal restrictions, also had a significant influence on children’s rights and safety in mental health care [23, 32, 38]. Inadequate refugee policies were seen by mental health professionals as a major cause of instability, mistrust and anxiety, which in turn had a negative impact on help-seeking and treatment outcomes [23]. Service providers also emphasised that existing policies lead to differences in the choice and quality of treatment compared to host country children [32].

Given the impact of policies and legal restrictions, mental health service providers emphasised that action to uphold the rights and safety of FDCA in mental health services should start at the policy level [23]. Other facilitators included the implementation of culturally sensitive informed consent models and the allocation of sufficient time for psychoeducation and building trust in therapy [26, 35, 36]. Efforts to address mental health stigma were also highlighted as a main facilitator. Strategies included being sensitive to young people’s preferred language about mental health care, avoiding the use of terms such as ‘mental illness’ and instead framing mental health services as a socially embedded offer that promotes personal growth and social integration [30, 37]. Provision of training and supervision for mental health professionals and interpreters was identified as necessary to ensure effective and culturally appropriate communication [27, 35]. Mental health professionals highlighted the benefits of adequately preparing interpreters by providing clear guidance on the purpose and content of therapy, using strategies ranging from the provision of written materials to in-depth training [27].

#### Family and community engagement

Overall, studies strongly emphasized the importance of strengthening family, peer, and community engagement to improve mental health outcomes for FDCA [25, 33–37]. However, service providers described mental health stigma and a lack of mental health awareness in the communities as great barriers to family and community engagement [23, 33, 34]. In addition, engaging fathers in particular was described as a challenge, as they were described as absent for cultural, work or other reasons [33].

Beyond the family, service providers highlighted the importance of active engagement and collaboration with schools and teachers [23–25]. Schools were recognised as central to early intervention, social integration and referral pathways, with teachers often trusted by young people and acting as a first point of contact for their mental health concerns [23–25]. However, service providers reported a lack of links and coordination between mental health and community services, including schools [23, 32]. The need for better coordination between mental health providers, schools, social workers and non-governmental organizations (NGOs) to meet the holistic needs of children and youth was emphasized [33, 38]. Organizing regular network meetings was described as best practice to foster engagement with community services [38]. To further improve family and community engagement, the implementation of mobile and community-based services was recommended. Providing mental health support directly in schools, asylum centres or familiar community settings helped to reduce practical barriers and social exclusion, and increase uptake of services [27, 34, 35].

#### Smooth transitions

Transitions between services and adherence to treatment were reported to be challenging. A study in Turkey found that refugee children and families received significantly less follow-up treatment than non-refugee patients [39]. Refugee patients were less likely to drop out of mental health services if they had ADHD or PTSD, while language proficiency and duration of resettlement did not influence follow-up rates [39]. Mental health service providers noted that fragmented services and uncoordinated referral pathways are common in the mental health care for FDCA, affecting smooth transitions [23, 27, 31]. While developing effective working relationships with key organisations was seen as a key principle, a lack of knowledge exchange and systematic collaboration between agencies was reported, regardless of the availability of resources in the mental health system [23]. Service providers acknowledged that their ability to make appropriate referrals and coordinate care is limited by a lack of awareness of available referral services and agencies [23]. High eligibility thresholds for referrals to mainstream child and adolescent mental health services were reported. Referrals of FDCA were often rejected because their descriptions of distress did not match the diagnostic language commonly used within the services [38]. Service providers noted that referral institutions needed additional training in addressing the mental health needs of asylum-seeking and refugee minors [32]. Navigating the mental health care system and continuation of care was further hindered by administrative barriers such as uncertainties about the cost coverage of care and frequent relocations due to asylum procedures and uncertainties [32, 34, 38].

To improve smooth transitions, service providers recommended establishing a central public health body and coordinating agencies to strengthen inter-agency work in mental health care for FDCA [23]. Mental health services implemented a range of different strategies to improve networking and collaboration, such as disseminating information about the service to reach potential referrers or providing regular training to network partners in trauma-informed approaches [27, 37]. In order to facilitate a clear understanding of referral and (re-)access processes for service users, providers described the usefulness of making information easily accessible, available in different languages, and culturally sensitive. In addition, having a follow-up mechanism in place helped to guide patients through the transition of services [23].

#### Timely support

Legal restrictions and uncertainties regarding children’s and adolescents’ asylum status were described as significantly delaying timely access to mental health services [23, 31, 32]. Despite presenting with severe trauma-related symptoms, many forcibly displaced youth did not receive timely assessments, leading service providers to believe that trauma-related disorders were systematically underdiagnosed within the mental healthcare system [32]. Waiting times to access mainstream mental health services were long, with specialised services often fully booked and unable to refer clients elsewhere due to a lack of other services providing therapy to refugee and asylum-seeking adolescents [32]. Service providers interviewed by Yim et al. [38] and Lampa et al. [27] similarly pointed out that the currently allocated resources and funding uncertainties for child and adolescent refugee mental health services were critical barriers to early intervention. In response, service providers advocated for short-term, cost-effective, and practical interventions [33, 34]. Mental health professionals emphasized the need to implement more scalable psychosocial interventions [34]. Task-shifting approaches, involving mental health and psychosocial support delivered by trained lay-professionals, were seen as beneficial for providing initial support and identifying at-risk children and adolescents at an early stage [33].

#### Developmentally appropriate and evidence-based

Mental health service providers interviewed by Yim et al. [38] described the use of a range of evidence-based interventions to treat unaccompanied asylum-seeking children. However, commissioners adapted their commissioning practices based on clinicians’ concerns with delivering trauma-focused treatment when unaccompanied asylum-seeking children were likely to return to their accommodation without support. Specifically, commissioners preferred to commission ‘light-touch therapeutic input’ from third sector providers rather than guideline-recommended and evidence-based trauma-focused therapies [38]. Michelson and Sclare [31] found that unaccompanied asylum-seeking minors were considerably less likely to receive cognitive therapy, anxiety management, and practical assistance for basic social needs compared to accompanied asylum-seeking minors. Rates of trauma-focused intervention provision did not differ between groups, despite significantly higher rates of PTSD among unaccompanied minors [31].

Service providers emphasised the integration of social, sports and arts activities to promote age-appropriate, flexible and engaging treatment [22, 26, 36]. Children and adolescents reported that such activities not only provided a sense of normalcy and social connection, but also facilitated building trust and understanding with mental health professionals [26]. Service providers also highlighted the challenges of providing developmentally appropriate care in mixed-aged groups [22]. Specific challenges included identifying suitable activities to engage teenagers and ensuring that younger children did not attend sessions designed for older age groups [22].

#### Competent and appropriate workforce

According to service providers, staff shortages and high staff turnover contributed to team instability and difficulties in communication and collaboration, negatively affecting treatment effectiveness and continuity of services [22, 32, 34]. A lack of clinical supervision was also cited, leading to hesitancy in managing complex cases [34].

Children and adolescents frequently mentioned a lack of cultural competence among service providers [26, 29]. Moreover, they perceived mismatches in gender, ethnic and cultural background, and interpersonal style with their therapists, with some discontinuing therapy because they no longer felt comfortable with their therapist [29, 32]. Carers also identified that more diverse representation in the workforce can facilitate building therapeutic relationships and treatment [29].

Training and ongoing supervision were identified as key facilitators for workforce competence. Service providers stressed the need for training focused on traumatic stress and to adapt mental health support to the needs of refugee children and young people [34, 37]. They also recommended training on strategies and skills to protect staff psychological well-being [34].

#### Quality improvement and data collection

Challenges to quality improvement and data collection were reported at both service and policy level. Patient-based treatment assessments faced several barriers, including dropout, time constraints and difficulties in administering questionnaires [35]. Some therapists were also reluctant to carry out mental health assessments fearing it might further distress young people [35]. Service providers pointed to the need to define indicators and parameters in order to formulate realistic service plans and to monitor and record their impact [33]. It was emphasised that indicators must be feasible to measure and easily integrated into the service [33]. At policy level, the mental health database was constrained by incomplete data that lacked key information on the asylum status of children receiving care [38].

## Discussion

This scoping review aimed to provide a comprehensive synthesis of the barriers and facilitators to quality mental health care for FDCA in the WHO European Region. The review included 18 studies, representing 7 of the 53 Member States in the WHO European Region. There was a lack of quantitative and mixed methods research, with the majority of studies based on qualitative research designs. Using the WHO Quality Standards for Child and Youth Mental Health Services as an analytical framework, the review identified relevant barriers and facilitators for quality improvement. The findings highlight the urgent need for policies that prioritise rights-based, evidence-based and child-centred approaches, as well as workforce strengthening, to improve the quality of mental health care for FDCA. Additionally, there is a need for more robust evidence and increased implementation of quality assessment and improvement in mental health care for FDCA throughout the WHO European Region.

Overall, the reviewed literature suggests that efforts are needed to improve quality of mental health care for FDCA in the WHO European Region, as indicated by quality deficiencies across all domains. Concerns about the quality of services were expressed by children and young people, carers, service providers, service coordinators and commissioners alike. Several studies indicated significant differences in the quality of mental health care received by FDCA compared to their non-displaced peers [32, 38, 39]. These findings are consistent with previous research on mental health care for adult refugees and asylum-seekers, which found that even comparatively well-resourced health systems fail to provide equitable and adequate mental health care for this patient population [13, 40]. While two thirds of countries in the WHO European Region have stand-alone mental health policies for children and adolescents [41], our findings suggest that these policies do not necessarily ensure equitable service provision for forcibly displaced youth. Moreover, within the European Union, the comprehensive approach to mental health emphasizes the need to protect children and young people, with specific commitments to addressing the needs of vulnerable populations and targeted support mechanisms for those most in need [42]. However, given the findings of this review, there remains a clear gap between policy commitments and actual service provision, often leaving FDCA underserved and without adequate mental health care.

There are multiple, intersecting barriers that impede the quality of mental health care for FDCA in the WHO European Region. Key barriers included restrictive policies and administrative barriers, service constraints and workforce competence gaps. These findings are consistent with previous reviews on access to mental health care for adult refugees and asylum seekers as well as migrant youth [17, 18, 40]. Access to care is often hindered by supply-side barriers, including structural deficiencies in mental health systems, restrictive policies and provider characteristics [17, 18, 40]. Our review shows that barriers pertaining to the health system not only impede access but undermine the overall quality of mental health care for forcibly displaced children and youth. This was particularly evident for restrictive policies and service provider competence, which were a major challenge to several quality domains, including participation and empowerment, rights and safety, timely support, and smooth transitions. Thus, extending previous research, our findings illustrate a double burden caused by structural and systemic barriers, with FDCA not only facing great difficulties in accessing the mental health care system, but often receiving suboptimal care when they do.

Key facilitators to quality mental health care included (a) integrating mental health support into everyday settings such as communities and schools, (b) strengthening networks of interconnected comprehensive services, (c) enhancing provider competencies in culturally sensitive care and treatment of trauma-related mental health problems, and (d) using task-shifting approaches and scaling up evidence-based psychosocial interventions. These findings are consistent with guidance on the design and delivery of high-quality, appropriate, and cost-effective mental health services for children and adolescents [43]. Thus, actively promoting the facilitators identified in this review may not only help to ensure that children and youth receive timely and appropriate care but also to optimize the use of available mental health resources.

Our review identified considerable gaps in the literature, particularly in geographical representation and research methodologies. A striking research gap is the limited availability of studies on the quality of mental health care for FDCA across many countries in the WHO European Region. The identified articles cover only seven of 53 countries in the WHO European Region, leaving 87% of member states without data. Notably, some of the seven countries represented in the literature are among the largest host countries for forcibly displaced populations in the WHO European Region. According to UNHCR data, Turkey and Germany together host approximately 2.3 million FDCA [15]. Collectively, the countries included in this review account for a substantial share of the FDCA population in the Region. However, several other countries with large FDCA populations - such as Ukraine, Poland, Azerbaijan, and Greece - are not represented in the literature. Moreover, the majority of available studies originate from a small number of Western European host countries, leaving other parts of the WHO European Region underrepresented. This not only limits generalizability but impairs regional equity in policy responses. It also reflects broader trends in refugee and migrant health research, which tends to concentrate on a few receiving countries rather than capturing the full migration journey [44]. Our review also highlights a lack of methodological diversity, with a predominant focus on qualitative research using semi-structured interviews and small sample sizes. Few of the studies used quantitative or mixed methods approaches to examine mental health care quality for FDCA. This aligns with broader patterns in refugee and migrant health research, which is often characterised by observational studies and small-scale research, with a lack of mixed-methods research or large comparative studies [45]. The WHO Framework for Refugee and Migrant Health Research recommends that information from a variety of study designs is crucial for building a comprehensive evidence base [45]. Overall, our findings suggest that more data is needed to provide an adequate basis for evidence-based decision making and quality improvement in mental health care for forcibly displaced children and youth.

### Limitations

As a scoping review, this review does not include a quality assessment of the included studies and does not provide definitive conclusions about the strength of the evidence. While the focus on studies published since 2004 captured recent research and trends in forced displacement, it may have excluded older studies with valuable information. The specific focus on the WHO European Region does not allow generalization of the results to other regions. Furthermore, although our review aimed to identify common barriers and facilitators to quality mental health care for all FDCA in the WHO European Region, caution should be exercised in generalizing the findings, as FDCA are a diverse population. Experiences in the mental health care system are likely to vary according to intersectional characteristics of those receiving care (e.g. gender, region of origin, religion) and characteristics of the health care system in different host countries (e.g. legislation, social norms).

### Implications for policy, practice, and research

Our findings emphasize that existing barriers should and can be addressed. However, the current policy landscape in the WHO European Region offers few facilitators for improving the quality of care. In particular, asylum policies that restrict access to health care continue to hinder the timely and efficient provision of mental health services to this population. This is despite the fact that untreated and poorly managed mental health problems lead to high and recurring costs for the treatment of chronic mental illness and emergency interventions in European countries [42]. Increased investments in the availability and quality of mental health services may contribute to improved educational outcomes, social participation and integration of forcibly displaced children and youth. At the same time, social integration and access to basic needs (e.g., housing, education, legal security, safety) can themselves be vital for mental health, emphasising the importance of policies that address the underlying structural determinants of health. To optimize the use of available resources in mental health care systems, community-based mental health services, relying on networks of interconnected services and stepped care and task-shifting approaches, are recommended and should be promoted throughout the region [43]. In using task-shifting, it is crucial to ensure that non-professional providers have the required competencies to deliver services safely and effectively [46]. As a roadmap for quality improvement, efforts to improve mental health care for FDCA can potentially be guided by the WHO Quality Standards for Child and Youth Mental Health Services, which emphasize equitable care [19]. Policies should focus on embedding quality standards within national mental health frameworks and ensuring that services are inclusive, participatory and adequate for all children and adolescents, including those affected by forced displacement.

While the facilitators identified in this review may provide a valuable starting point for addressing quality challenges, actively implementing quality improvement methods including systematic data collection and monitoring is recommended. Moreover, young people should be actively involved in quality improvement efforts to ensure that their perspectives, preferences, and lived experiences are reflected in mental health care. Recent publications provide valuable guidance on youth participation in mental health care [47, 48]. An additional important means for quality improvement is capacity building of service providers. Specifically, training to increase the capacity of professionals to work with forcibly displaced children and youth should be incorporated into curricula at an early stage. This may include increasing knowledge of the diversity of needs, the ability to build trustful therapeutic relationships, non-discriminatory and culturally sensitive practice, and the ability to work with other agencies, support networks, and interpreters.

The limited availability of research and data on the quality of mental health care for FDCA highlights the urgent need for more rigorous and comprehensive studies in this field. In line with the WHO Framework for Refugee and Migrant Health Research in the WHO European Region [45], future research should employ a broader range of methodologies, including mixed methods approaches, comparative designs, and participatory studies to identify key areas for quality improvement. Furthermore, the use of measurable quality indicators and the establishment of baseline data may serve as a foundation to systematically evaluate quality improvement efforts and identify strategies. Therefore, WHO European Region member states should routinely collect quality health data disaggregated by migration status, age, and gender to inform a responsive health policy [4]. This may be particularly important in countries that are currently underrepresented in the literature. Expanding research to all member states is essential to develop a context-specific understanding of mental health service provision and identify appropriate quality improvement strategies. Understanding which approaches work best, in which contexts, and for whom will be crucial for guiding service development and policy reforms towards high-quality mental health care.

## Conclusion

This scoping review provides a comprehensive synthesis of barriers and facilitators to quality mental health care for FDCA in the WHO European Region. Using the WHO Quality Standards for Child and Adolescent Mental Health Services as an analytical framework, we identified deficiencies across all quality domains, with restrictive policies, administrative barriers, workforce constraints, and a lack of culturally responsive and child-centred care emerging as key barriers. Important facilitators that can enhance care quality include community-based care models, strengthened multi-agency collaboration, enhanced provider competencies, and the scaling up of evidence-based interventions. Major gaps remain in the literature on the quality of mental health care for FDCA in Europe, as research is concentrated in a limited number of countries and lacks methodological diversity. Quality improvement and policy changes are needed to ensure that FDCA receive adequate mental health care.

## Supplementary Information

Below is the link to the electronic supplementary material.

ESM 1 (PDF 56.7 KB)

## Data Availability

No datasets were generated or analysed during the current study.

## Footnotes

1 We refer to forcibly displaced populations in accordance with definitions provided by the United Nations High Commissioner for Refugees (UNHCR), as individuals and communities who have been forced to leave their homes due to conflict, violence, human rights violations, or events seriously disturbing public order. This may include asylum-seekers (those who have crossed an international border and applied for international protection, with claims for international protection status not yet determined), refugees (those granted international protection status after crossing a border), as well as internally displaced persons (those displaced within the borders of their home country).
