# Supplementary material for: Quality of mental health care for forcibly displaced children and adolescents in the WHO European region: A scoping review of barriers and facilitators
Source: Eur Child Adolesc Psychiatry. 2025 Aug 27;35(1):75–90. doi: 10.1007/s00787-025-02833-3 (PMC12917058; doi:10.1007/s00787-025-02833-3)
Supplement: Supplementary file 1 — (PDF 56.7 KB) [file 787_2025_2833_MOESM1_ESM.pdf]

Online Resource 1

WHO Quality Standards for Child and Youth Mental Health Services

Quality Theme	Definition
1. Participation and Empowerment	Active participation of children, young people, and caregivers in service planning and implementation, and empowerment to make informed decisions about their care.
2. Rights and Safety	Safe, respectful, and rights-based care that protects all individuals from harm and ensures equitable, non-discriminatory inclusion of all children, young people, and their caregivers.
3. Family and Community Engagement	Involving families in care where appropriate and building strong partnerships with community organizations to support the holistic needs of children, young people, and their caregivers.
4. Smooth Transitions	Coordinated and collaborative referrals and transitions between services that ensure continuity of care, particularly for those at risk of harm.
5. Timely Support	Early and timely assessment and intervention for children, young people, and their caregivers.
6. Developmentally Appropriate and Evidence-Based	Provision of evidence-based care tailored to the developmental stage, needs, and preferences of children, young people, and their families.
7. Competent and Appropriate Workforce	A well-trained, adequately staffed workforce with the skills and support needed to build strong therapeutic relationships and deliver high-quality care.
8. Quality Improvement and Data Collection	Ongoing monitoring, evaluation, and governance to improve service quality, supported by adequate resources for data collection, analysis and reporting.

Note. Adapted from: World Health Organization. Regional Office for Europe. (2025). Quality standards for child and youth mental health services: for use in specialized community or outpatient care across the WHO European Region. World Health Organization. Regional Office for Europe. https://iris.who.int/handle/10665/380778.
